# Two novel myocilin mutations in a Chinese family with primary open-angle glaucoma

**Published:** 2008-09-05

**Authors:** Xiaobing Xie, Xin Zhou, Xiying Qu, Jing Wen, Yanli Tian, Fang Zheng

**Affiliations:** 1 Center for Gene Diagnosis, Zhongnan Hospital, Wuhan University, Wuhan, China; 2 Renmin Hospital, Wuhan University, Wuhan, China

## Abstract

**Purpose:**

To investigate the genetic linkage of primary open-angle glaucoma (POAG) in a Chinese family.

**Methods:**

We have screened for myocilin (*MYOC*) gene mutations in a glaucoma family of five generations. There are fifty-six members of whom 11 were confirmed to have POAG , two with ocular hypertension were considered as POAG suspect, and the remaining 43 were asymptomatic. We also recruited 200 unrelated healthy Chinese subjects as normal control. Polymerase chain reaction-restriction fragment length polymorphism (PCR-RFLP) analysis and DNA sequencing were used to identify mutations in the three exons of *MYOC*. Presymptomatic diagnoses were made for the family members seeking consultation based on the results of both clinical examination and genetic analysis.

**Results:**

Among three allelic variants identified in this pedigree (Pro13Leu [38 C**→**T], Arg76Lys [227G**→**A], and Gln337Stop [1009C del]), Pro13Leu and Gln337Stop were reported to be novel mutations while Arg76Lys has been previously documented. Our results show that all 11 POAG patients carry the Gln337Stop mutation and that four POAG patients and one POAG suspect (V:2) were found to have the Pro13Leu mutation. In addition, Arg76Lys polymorphism was identified in two patients and a POAG suspect (V:5).

**Conclusions:**

Pro13Leu and Gln337Stop mutations of *MYOC* are likely responsible for the etiology of POAG in this pedigree, but the causative mechanism needs further research.

## Introduction

Glaucoma is the second leading cause of blindness worldwide, affecting more than 70 million people. Primary open-angle glaucoma (POAG) is the most common form of this ocular disease. A population-based, cross-sectional study showed that glaucoma is the major cause of blindness in China, and POAG is a key form of the disease [1]. Noticeably, incidence rates of secondary glaucoma and congenital glaucoma are 0.52% and 0.02%, respectively. The prevalence of primary glaucoma is 1%-2% in the population over age 40.

POAG is usually asymptomatic until the late stage of the disease. This make early diagnosis almost impossible. And when POAG reaches the late stage, irreversible damages such as chronic, progressive apoptosis of optic ganglion cells and visual field damage usually occur. The most important risk factor for POAG is family history [2]. First-degree relatives of individuals affected with POAG are 10 times more likely to develop POAG [3]. Since its first implication in the genetic linkage to POAG in 1997, numerous mutations in the myocilin (*MYOC*) gene have been identified and their specific phenotypes have been characterized. Although the mechanism underlying glaucoma is poorly understood, a growing body of evidence suggests that there is a genetic link between *MYOC* mutations and the pathogenesis of glaucoma. So far, more than 70 mutations of *MYOC* have been documented in POAG families or sporadic POAG patients. Some of them such as Gln48His [4] in exon 1, Asp208Glu [5] in exon 2, and Pro370Leu [6] and Thr377Met [7] in exon 3 of *MYOC* were confirmed to correlate with POAG. Interestingly, *MYOC* mutations have been found to vary with different ethnic groups and geographic locations [8-14]. In the current study, we performed *MYOC* mutation screening in a large glaucoma family affected with POAG, and our results suggest that novel mutations of *MYOC*, Pro13Leu and Gln337Stop, may be associated with POAG. This study will also discuss the significance of our findings to genetic counseling.

## Methods

### Clinical examination and diagnosis of primary open-angle glaucoma

Clinical examinations were performed including visual acuity, slit lamp biomicroscopy, applanation tonometry, gonioscopy, funduscopy, and perimetry. Family members were divided into three groups: (1) affected individuals, (2) asymptomatic individuals, and (3) suspect individuals. POAG is defined by a normal appearing anterior chamber angle along with two of the following symptoms [15]: elevation of intraocular pressure (IOP>21 mmHg), characteristic visual field defects, and glaucomatous optic nerve head changes (cup-disc ratio>0.6 or notches). Subjects meeting only one of these symptoms were defined as suspect. Individuals without any manifestations were defined as asymptomatic. Patients were diagnosed and treated at the Eye and ENT Hospital (Fudan University, Shanghai, China). All participants in this research had given informed consent after receiving detailed explanation of the nature and possible consequences of the study.

### Genetic analysis

Genomic DNA was extracted from peripheral blood leukocytes using standard procedures. The coding sequences of *MYOC* (GenBank AB006688) were amplified by polymerase chain reaction (PCR). Amplifications of three exons were performed in a 25 μl reaction containing 50 ng of genomic DNA mixed with 10X buffer, 50 pmol primers, 2.5 mM NTP, and 1 ul Taq polymerase. PCR conditions were as follows: initial denaturation at 94 °C for 5 min followed by 35 cycles of denaturation at 94 °C for 45 s, annealing at a temperature specific for each primer for 45 s (Table 1), and extension at 72 °C for 1 min. A final extension at 72 °C for 10 min completed the reaction. The reactions were performed with a GeneAmp PCR system 9600 (Applied Biosystems, Foster City, CA). Subsequently, 8 μl of the PCR product for each polymorphic site was digested completely with Bme1390 I, BselI, Eco721, MspI, Bpu11021, PagI, BsmaI, and BseD, according to instructions recommended by the manufacturer. Genotype analysis was determined by 12% polyacrylamide gel. Direct sequencing was performed on an Applied Biosystems 3730 DNA Analyzer (Applied Biosystems) according to the BigDye Terminator version 3.1 protocol. *MYOC* mutations were screened in each surviving individual. Presymptomatic genetic diagnoses were determined for family members who sought information and instruction about their disease status according to the phenotype of POAG, the pattern of inheritance, their clinical status, and genetic analysis results of their family. Follow-up plans were established after these diagnoses.

**Table 1:** Primers and sequences used in this study.

**ID**	Position*	**Tm** **(°C)**	**Sequence (5′→3′)**	**Size (bp)**
MYOC1	353–509	62	F: GGCTGGCTCCCC AGTATATA	174
			R: ACAGCTGGCATCTCAGGC	
MYOC2	491–658	62	F: ACG TTG CTG CAG CTT TGG	196
			R: GATGACTGACATGGCCTGG	
MYOC3	603–772	65	F: AGTGGCCGATGCCAGTATAC	189
			R: CTGGTCCAAGGTCAATTGGT	
MYOC4	670–864	62	F: AGGCCATGTCAGTCATCCAT	214
			R: TCTCTGGTTTGGGTTTCCAG	
MYOC5	778–958	60	F: TGACCTTGGACCAGGCTG	200
			R: CCTGGCCAGATTCTCATTTT	
MYOC6	928–1095	63	F: TGGAGGAAGAGAAGAAGCGA	187
			R: CTGCTGAACTCAGAGTCCCC	
MYOC7	1416–1630	62	F: AACATAGTCAATCCTTGGGCC	230
			R: TAAAGACCATGTGGGCACA	
MYOC8	1933–2091	60	F: TTATGGATTAAGTGGTGCTTCG	177
			R: ATTCTCCACGTGGTCTCCTG	
MYOC9	2069–2232	64	F: AAGCCCACCTACCCCTACAC	184
			R: AATAGAGGCTCCCCGAGTACA	
MYOC10	2195–2366	64	F: ATACTGCCTAGGCCACTGGA	190
			R: CAATGTCCGTGTAGCCACC	
MYOC11	2335–2512	63	F: TGGCTACCACGGACAGTTC	197
			R: CATTGGCGACTGACTGCTTA	
MYOC12	2480–2653	64	F: GAACTCGAACAAACCTGGGA	195
			R: CATGCTGCTGTACTTATAGCGG	
MYOC13	2624–2783	62	F: AGCAAGACCCTGACCATCC	179
			R: AGCATCTCCTTCTGCCATTG	

The asterisk indicates that all numbers correspond to the numbering scheme of GenBank AB006688

### Secondary structure prediction

We also used Antheprot software to analyze the possible effects of these mutations on the secondary structure of the corresponding proteins.

## Results

### Phenotypes of the patients

**Figure 1:**
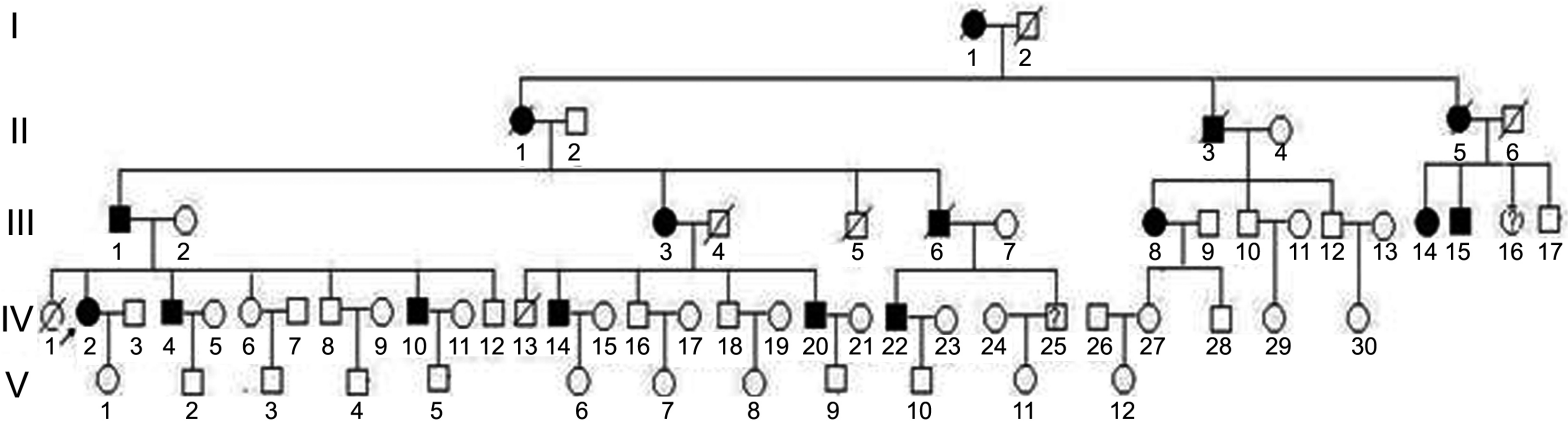
Pedigree with variances of *MYOC.* Solid symbols and open symbols represent affected and unaffected disease status, respectively. Suspects are marked with a question mark inside the squares. Roman numerals and Arabian numerals indicate generations and orders, respectively. Squares and circles represent males and females, respectively. A slash indicates a deceased family member. The arrow indicates the proband.

The five-generation family exhibited an autosomal dominant pattern of inheritance (Figure 1). A total of 11 patients were identified with POAG (Table 2). The information for III:8, III:14, and III:15 were not complete. Their diagnoses were based on medical histories, which included elevation of intraocular pressure (IOP >21 mmHg) and characteristic visual field defects.

**Table 2:** Clinical appearance of the affected members

**Family member**	**Sex**	**Age (years)**	**Age of onset (years)**	**Visual acuity**	**Maximum IOP (mmHg)**	**Cup-disc ratio**	**VF grading score**	**Severity**	**Medical therapy**
III:1	M	66	23	OD: NLP	46	**	#	End stage	Surgery##
				OS: NLP	44	**	#	End stage	Surgery
III:3	F	63	24	OD: NLP	*	**	#	End stage	Surgery
				OS: NLP	*	**	#	End stage	Surgery
III:8	F	56	26	OD: NLP	*	**	#	End stage	Surgery##
				OS: NLP	*	**	#	End stage	Surgery
III:14	F	38	21	OD: NLP	*	**	#	End stage	Surgery
				OS: NLP	*	**	#	End stage	Surgery
III:15	M	35	19	OD: NLP	*	**	#	End stage	Surgery
				OS: NLP	*	**	#	End stage	Surgery
IV:2	F	42	41	OD: 0.5	28	0.7	1	Mild	No
				OS: 0.4	22	0.6	2	Mild	No
IV:4	M	36	26	OD: NLP	*	**	#	End stage	Surgery
				OS: NLP	*	**	#	End stage	Surgery
IV:10	M	30	22	OD:0.01	29	1	8	Severe	Surgery
				OS: NLP	28	1	#	End stage	Surgery
IV:14	M	40	16	OD: NLP	29	0.8	#	End stage	No
				OS: NLP	32	0.8	#	End stage	No
IV:20	M	33	26	OD: NLP	28	**	#	End stage	No
				OS: NLP	*	**	#	End stage	Surgery
IV:22	M	35	30	OD: 1.0	18	0.4	0	Mild	No
				OS: 0.25	35	0.6	3	Moderate	No

The asterisk indicates that the maximum intraocular pressure (IOP) was not certain because of relatively advanced glaucoma at the time of first presentation. The double asterisk indicate that the cup/disc ratio was not certain because of eyeball atrophy or cornea opacity. The sharp (hash mark) indicates that the visual field was not available because the patient had no light perception. The double sharp (hash marks) indicates that the member had surgery twice. The visual fields (VF) were graded for severity of visual field loss with a level grading scale. Severity classification is leveled by the visual field score: mild (0–2), moderate (3–5), severe (6–8), and end stage (no light perception). NLP, no light perception.

**Figure 2:**
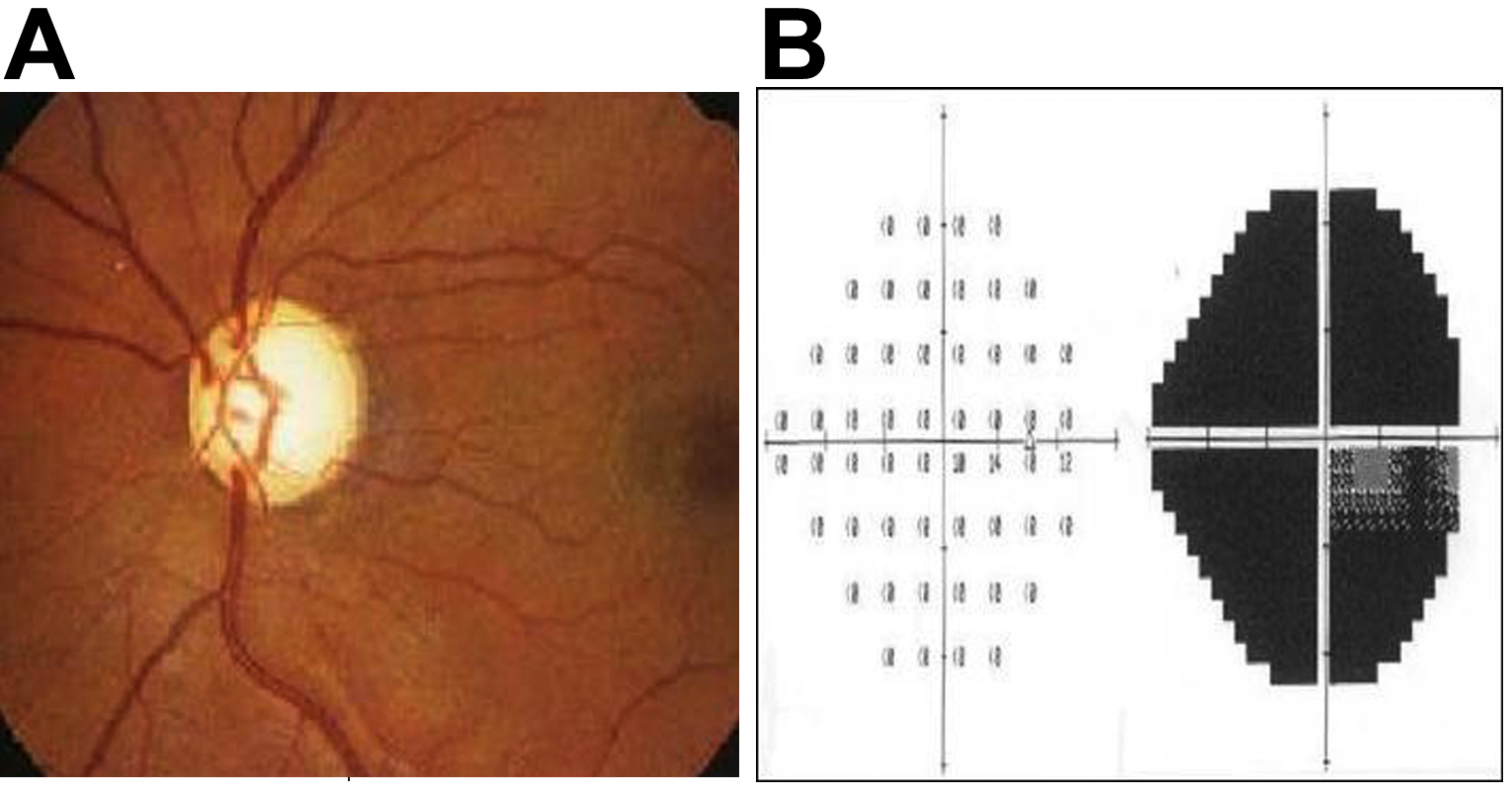
Optic disc and visual field of individual IV:22. Glaucomatous optic disc atrophy is seen with visual field defects in the right eye. Panel **A** shows the optic disc atrophy of the right eye while panel **B** shows visual field defects of the right eye.

Onset ages of patients ranged from16 to 41 years. Most patients had typical glaucoma changes in the optic disc and the visual field (Figure 2). All patients showed more damage to the optic nerve head in the right eye than in the left. All affected family members had a noticeable increase in intraocular pressure (IOP) higher than 22 mmHg. The IOP of these patients could not be controlled with available antiglaucoma medication. Most of the patients underwent antiglaucoma surgeries, and subjects III:1 and III:8 had repeated operations due to the failure of the first procedure but still couldn’t control the development of the pathogenetic condition.

### Clinical examination of the consulters

There were altogether 26 consulters with no visible optic head damage and visual field defects (Table 3), and two of whom (III:16 and IV:25) were marked as suspects since their maximum IOP was higher than 21 mmHg. No evidence for glaucoma was found in the other 24 consulters.

**Table 3:** Clinical features and gene screening results of the consulters.

**Family member**	**Sex**	**Age (years)**	**Maximum IOP (mmHg)**	**Cup-disc ratio (OD/OS)**	**Visual field score (OD/OS)**	**Diagnosis**
III:10	M	54	19	0.3/0.4	0/0	Unaffected
III:12	M	51	20	0.5/0.4	0/0	Unaffected
III:16	F	30	21	0.4/0.4	0/0	Suspect
III:17	M	27	18	0.4/0.3	0/0	Unaffected
IV:6	F	34	17	0.4/0.3	0/0	Unaffected
IV:8	M	32	15	0.3/0.2	0/0	Unaffected
IV:12	M	25	19	0.3/0.3	0/0	Unaffected
IV:16	M	38	20	0.5/0.5	0/0	Unaffected
IV:18	M	36	20	0.5/0.5	0/0	Unaffected
IV:25	M	35	21	0.4/0.3	0/0	Suspect
IV:27	F	30	15	0.4/0.3	0/0	Unaffected
IV:28	M	25	19	0.3/0.3	0/0	Unaffected
IV:29	F	28	16	0.3/0.2	0/0	Unaffected
IV:30	F	24	18	0.4/0.3	0/0	Unaffected
V:1	F	19	18	0.4/0.3	0/0	Unaffected
V:2	M	13	16	0.3/0.5	0/0	Unaffected
V:3	M	11	17	0.4/0.5	0/0	Unaffected
V:4	M	9	16	0.4/0.4	0/0	Unaffected
V:5	M	9	16	0.5/0.5	0/0	Unaffected
V:6	F	16	17	0.4/0.3	0/0	Unaffected
V:7	F	15	18	0.4/0.4	0/0	Unaffected
V:8	F	13	20	0.4/0.3	0/0	Unaffected
V:9	M	10	18	0.4/0.3	0/0	Unaffected
V:10	M	4	20	0.5/0.5	0/0	Unaffected
V:11	F	12	19	0.2/0.4	0/0	Unaffected
V:12	F	8	17	0.3/0.4	0/0	Unaffected

### Mutation screen of *MYOC*

**Figure 3:**
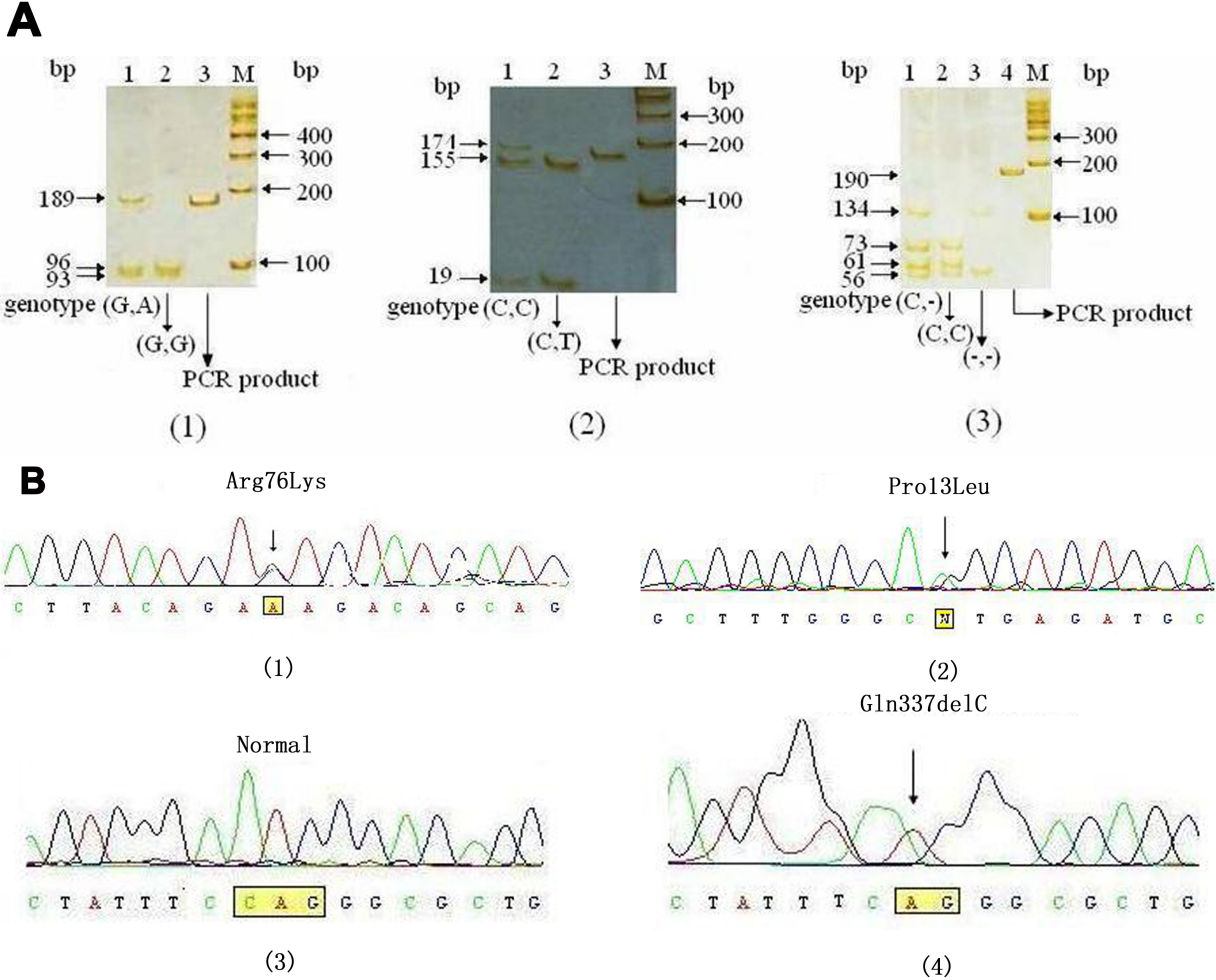
Electrophoretic mobility assays of PCR-RFLP and sequence results. **A**: PCR products were separated on 12% polyacrylamide gel from three representative samples, and the genotypes are shown for (1) Arg76Lys (227G**→**A), (2) Pro13Leu (38 C**→**T), and (3) Gln337Stop (1009C del). The sizes of the molecular weight marker and DNA fragments are shown in the left and the right sides of the gel, respectively. **B**: The representative chromatogram contains the sequence from the mutant DNA sequence strand for (1) Arg76Lys and (2) Pro13Leu as well as from the (3) wild-type and (4) mutant DNA sequence of Gln337Stop.

With polymerase chain reaction-restriction fragment length polymorphism (PCR-RFLP) and gene sequencing technologies, we identified one known mutation, Arg76Lys (227G**→**A), that was reported as a polymorphism by Alward et al. [15], and two novel mutations, Pro13Leu (38 C**→**T) and Gln337Stop (1009C del), that are likely responsible for the pathogenesis of POAG since these mutations result in either a change in the amino acid sequence or a frame shift. The three mutations were summarized in Table 4. The results of PCR-RFLP and gene sequencing are shown in Figure 3. Other mutations i.e., Gln48His (144G**→**T), Gly246Arg (736G**→**A), Gln337Arg (1009C**→**G), Ile345Met (1036 A**→**G), Pro370Leu (1109C**→**T), Asp380Asn (1138G**→**A), Asp380Ala (1139A**→**C), Ile477Ser (1430T**→**A), Pro481Thr (1441C**→**A), and Pro481Leu (1442C**→**T), were not detected.

**Table 4:** Summary of *MYOC* mutations found in this study.

**Mutation**	**Pedigree members**	**Controls**	**Reference**
Arg76Lys (227G**→**A)	3/56 (5.3%)	0	[15]
Pro13Leu (38 C**→**T)	5/56 (8.9%)	0	Present study
Gln337Stop (1009C del)	12/56 (21.4%)	0	Present study

### Presymptomatic diagnosis for consulters

Of the 26 consulters, two (III:16 and IV: 25) were classified as suspects because their maximum IOPs were higher than 21 mmHg. Another two (V:2 and V:10) at high risk for POAG since they carried the mutation considered to be disease-causing. One adolescent (V:2) carrying the Arg76Lys (38 C**→**T) mutation and another adolescent (V:10) carrying the Gln337Stop (1009 C del) mutation were defined as preclinical status and as having a high risk of developing glaucoma. To prescribe appropriate medication to carriers at an early stage of glaucoma, follow-up plans were established. The adolescents were asked to accept applanation tonometry and funduscopy every month and perimetry every six months.

### Prediction of two-dimensional structure

Protein analysis using Antheprot suggested that Pro13Leu (38 C**→**T) and Arg76Lys (227 G**→**A) mutations resulted in the modification of the corresponding amino acid, but the predicted secondary structures of the encoded proteins were not different from those of the wild-types (Table 5). However, the frame shift introduced by Gln337stop (1009C del) creates a premature termination codon thereby resulting in a truncated product.

**Table 5:** Antheprot analysis results of the wild-type and mutant protein comparison using different secondary structure prediction methods.

**Methods**	***MYOC* mutations**	**Alpha** **Helix (%)**	**Sheet** **(%)**	**Beta turn Random (%)**	**Coil (%)**
GOR	Wild-type	36	23	19	22
	Arg76Lys (227G->A)	36	23	19	22
	Pro13Leu (38 C->T)	36	23	20	21
	Gln337Stop (1009C del)	36	20	20	24
DMP	Wild-type	38	28	9	25
	Arg76Lys (227G->A)	38	28	9	25
	Pro13Leu (38 C->T)	39	28	9	24
	Gln337Stop (1009C del)	42	24	10	24
PRD	Wild-type	39	13	0	47
	Arg76Lys (227G->A)	39	13	0	47
	Pro13Leu (38 C->T)	39	13	0	47
	Gln337Stop (1009C del)	44	11	0	45

The abbreviations in the “Method” column are; DPM: Double Prediction Method [23], PRD: Predator method [24], GOR: Garnier, Osguthorpe, Robson [25].

## Discussion

*MYOC* consists of three exons separated by two introns and encodes a protein of 504 amino acids. Myocilin is a secreted, 55–57 kDa glycoprotein that forms dimers and multimers and has several characteristic structural motifs including a myosin-like domain, a leucine zipper region, and an olfactomedin domain [16]. More than 70 mutations in *MYOC* have been reported, 90% of which occur within exon 3. Mutations in *MYOC* are found in 3.86% of Caucasian patients with POAG, including normal tension glaucoma or ocular hypertension, 3.30% of patients of African descendants including African Americans and black residents in Africa, and 4.44% of Asian patients [17].

We have screened a Chinese POAG family for *MYOC* base-pair variants and identified three allelic variants, Pro13Leu (38 C**→**T), Arg76Lys (227G**→**A), and Gln337Stop (1009C del). Since the Arg76Lys mutation in POAG was first reported in 1998 [15], numerous studies have investigated the role of *MYOC* in the etiology of POAG in various ethnic groups and found that the mutation rate of *MYOC* ranges from 12% to 18% [18-22]. In contrast to 0% in normal controls, a mutation frequency of 5.3% (3/56) in Arg76Lys was identified in the present study, which is lower than previously reported. Moreover, these mutations do not result in significant alterations in either the predicted secondary structure or the physico-chemical property. Of course, more samples should be collected and analyzed to draw a conclusion to precisely represent the data generated from the present study.

Here, we have identified two novel mutations, Pro13Leu (38C**→**T) and Gln337stop (1009 C del), in prevalence rates of 8.9% and 21.4%, respectively, in this family. POAG in one patient carrying the Pro13Leu mutation eventually developed into blindness. One subject (IV:25; without the Pro13Leu mutation) who showed a normal appearance without visible optic head damage and visual field defects had an IOP of 21 mmHg. Whether this switch of amino acid residue has a dominant negative effect remains unknown and must be further studied. The other mutation, Gln337stop, was identified in all clinically confirmed patients and an asymptomatic subject V:10 (Gln337stop genotype frequency: 21.4%, 12/56). In this family, Gln337stop was shown to be one of the most severe and common gene defects that result in POAG. The present study reveals that the Gln337stop mutation of *MYOC* is deemed to largely contribute to the early onset glaucoma in this pedigree. Median onset age of affected individuals with Gln337stop was 24.9 years. Nearly all patients had to accept surgery due to intraocular pressure. In addition, the one suspect (V:10) harboring the Gln337stop mutation was only four years old but had a high IOP and cup-disc ratio, which made it highly likely to be predisposed to POAG. Genetically, the loss of cytosine at 1009 creates a premature stop codon. The resulting truncated product may have a severe dominant negative effect, which plays a critical role in etiology of POAG.

Ethnic difference in the frequency and types of *MYOC* mutations among patients with POAG has been examined by case control studies. At present, screening tests in whole populations for *MYOC* defects are not feasible due to the low prevalence of *MYOC*-associated glaucoma. However, people at high risk of developing glaucoma may benefit from genetic testing, especially in early onset type of glaucoma pedigrees.

In summary, mutations in *MYOC* strongly correlate with the pathogenicity of POAG. Future studies on glaucoma will be focused on developing an early genetic diagnosis. Such a test will allow evaluating the penetrance of *MYOC* and bypassing limitations of the present clinical based methods, thus providing a chance to prevent the irreversible damages.
